# Association between antiphospholipid antibodies, rheumatic-immune inflammation, and coronary in-stent restenosis

**DOI:** 10.3389/fcvm.2025.1656305

**Published:** 2026-01-14

**Authors:** Wenxing Mao, Zhiming Wu, You Wei, Gaofeng Wang, Ting Xiong, Pan Chang, Fei Ye

**Affiliations:** Department of Cardiology, Nanjing First Hospital, Nanjing Medical University, Nanjing, Jiangsu, China

**Keywords:** antiphospholipid antibodies, interleukin-6, immune inflammatory profiling, in-stent restenosis, drug-eluting stents

## Abstract

**Background:**

Antiphospholipid antibodies (aPL) and systemic rheumatic-immune inflammation (RII) may be associated with angiographic in-stent restenosis (ISR) after drug-eluting stent (DES) implantation. We prospectively evaluated these associations in a large Chinese cohort of DES recipients.

**Methods:**

In this prospective cohort, we enrolled 2,503 consecutive adults who received at least one new-generation DES between May 2022 and January 2024. Preprocedural blood samples were assessed for antiphospholipid antibodies (anticardiolipin IgG/IgM, anti-β2-glycoprotein I IgG/IgM, and lupus anticoagulant) and inflammatory biomarkers [RII; high-sensitivity C-reactive protein (hs-CRP), interleukin-6 (IL-6), tumor necrosis factor-α (TNF-α), erythrocyte sedimentation rate (ESR), complement C3/C4, rheumatoid factor, and anti-cyclic citrullinated peptide (anti-CCP) antibodies]. At 12 months, invasive coronary angiography or coronary CT angiography (CCTA) was used to assess ISR. All ISR analyses were restricted to participants who completed 12-month imaging (the imaging-complete cohort). Multivariable logistic regression adjusted for prespecified clinical/lesion/stent covariates and imaging modality. Model performance was compared for a Clinical model vs. an immune-enhanced model (clinical  +  aPL  +  IL-6) with internal bootstrap validation. ICA-only analyses were prespecified. Clinically driven target-lesion revascularization (TLR) was evaluated with Cox models in all enrolled patients.

**Results:**

Of the 2,503 enrolled participants, 2,388 completed 12-month imaging, with ISR occurring in 193 participants (8.1%; 95% CI 6.9–9.1). In adjusted analyses, any aPL positivity (OR 1.92, 95% CI 1.34–2.74) and IL-6 (per doubling) (OR 1.25, 95% CI 1.10–1.42) were independently associated with ISR. Adding aPL and IL-6 improved discrimination (AUC 0.79 vs. 0.72, Δ  =  0.07, *p*  =  0.008), calibration, and reclassification (categorical NRI 0.18, integrated discrimination improvement (IDI) 0.04). Optimism-corrected AUC was 0.78. The findings were consistent in the ICA-only cohort (aPL OR 1.95; IL-6 OR 1.27). Over 12 months, TLR occurred in 100/2,503 (4.0%). aPL positivity (HR 2.08, 95% CI 1.36–3.18) and IL-6 (per doubling; HR 1.29, 95% CI 1.11–1.50) were associated with higher TLR risk.

**Conclusion:**

Baseline aPL seropositivity and higher IL-6 were associated with 12-month ISR and clinically driven TLR. Incorporating these immune markers improves risk discrimination beyond clinical and angiographic factors. External validation and interventional studies are warranted.

## Introduction

In-stent restenosis (ISR) is the re-narrowing of a previously stented coronary segment, often presenting with recurrent angina or acute coronary syndrome (1, 2). Although new-generation drug-eluting stents (DES) have lowered risk, ISR still occurs in ∼5%–10% of cases worldwide (3, 4), partly because contemporary percutaneous coronary intervention (PCI) increasingly treats diffuse disease and small vessels (5, 6). Management typically involves drug-coated balloons or repeat DES, with intravascular imaging guiding lesion-specific therapy (6, 7). ISR carries substantial consequences, including recurrent symptoms and the need for repeat PCI or coronary artery bypass grafting (CABG) (8).

Traditional clinical and angiographic predictors—such as diabetes, complex lesions, and long or small stents—show limited ability to discriminate against ISR risk (9). Biologically, ISR reflects an immune inflammatory response to vascular injury: endothelial denudation, vascular smooth-muscle proliferation, and extracellular matrix deposition drive neointimal hyperplasia and, later, neoatherosclerosis (2, 10). Systemic inflammation, indexed by elevated high-sensitivity C-reactive protein (hs-CRP) and interleukin-6 (IL-6), has been associated with restenosis and stent failure (10). Consistent with this, higher ISR rates have been reported in antiphospholipid syndrome, systemic lupus erythematosus (SLE), and rheumatoid arthritis, underscoring immune mechanisms in ISR (10). Circulating rheumatic-immune inflammation (RII) biomarkers, including hs-CRP, IL-6, TNF-α, and complement C3/C4, capture systemic immune activation, but their prognostic performance for stent outcomes remains inconsistent across studies (11).

Antiphospholipid antibodies (aPL), including anticardiolipin (aCL), anti-β2-glycoprotein I (anti-β2GPI), and lupus anticoagulant (LA), are central to antiphospholipid syndrome (APS). By engaging endothelial cells, platelets, and monocytes, aPL induce endothelial activation and amplify proinflammatory and procoagulant signaling (e.g., TNF-α, TGF-β, and p38 MAPK), producing a prothrombotic milieu (12, 13). They also activate complement and the mTOR pathway, further increasing thrombotic risk (11, 14). Clinically, aPL positivity is associated with arterial and venous thrombosis and atherosclerotic cardiovascular disease (15), and small studies suggest higher in-stent restenosis (ISR) rates among aPL-positive patients (16).

A major knowledge gap remains regarding the prognostic role of aPL and a comprehensive RII panel in contemporary DES recipients. We therefore asked whether immune inflammatory status at PCI can refine risk stratification for coronary ISR. Specifically, we will prospectively test whether baseline aPL positivity and an expanded RII biomarker panel independently predict angiographic ISR 12 months after implantation of new-generation DES. Secondary aims are to examine associations with clinically driven target-lesion revascularization (TLR), quantify the incremental predictive value of adding immune markers to established clinical models, and explore whether IL-6 statistically mediates any association between aPL and ISR.

## Methods

### Study design and setting

We conducted a single-center, prospective, observational cohort study at Nanjing First Hospital, Nanjing Medical University. Consecutive eligible adults undergoing PCI with implantation of at least one new-generation drug-eluting stent (DES) were enrolled between May 2022 and January 2024 and followed for 12 months. The protocol complied with the Declaration of Helsinki and was approved by the institutional ethics committee. All participants provided written informed consent before any study procedures.

### Participants

*Inclusion criteria*: (1) age ≥18 years old; (2) successful implantation of ≥1 new-generation DES (everolimus-, sirolimus-, or zotarolimus-eluting) for a *de novo* native coronary lesion; (3), ability and willingness to undergo scheduled 12-month invasive coronary angiography (ICA) or contrast-enhanced coronary CT angiography (CT-CA); (4) provision of written informed consent.

*Exclusion criteria*: (1) cardiogenic shock or mechanical circulatory support at the index PCI; (2) life expectancy <12 months; (3) chronic infection or active malignancy receiving systemic therapy; (4) estimated glomerular filtration rate <30 mL min^−1^·1.73 m^−2^ (unless on chronic dialysis); (5) chronic systemic corticosteroid or non-autoimmune biologic/immunosuppressant therapy; (6) pregnancy; (7) inability to comply with follow-up.

Patients with established autoimmune diseases (e.g., antiphospholipid syndrome, SLE, and RA) were not excluded. Diagnosis and ongoing treatment were recorded for covariate adjustment.

### Index PCI and baseline data

Before PCI, demographics, cardiovascular risk factors, prior MI/PCI/CABG, presentation [acute coronary syndrome vs. stable coronary artery disease (CAD)], and medications were captured in an electronic case report form. Procedural variables [target vessel, American College of Cardiology/American Heart Association (ACC/AHA) lesion class, SYNTAX score, stent platform/number/length/diameter, intravascular imaging use, and post-dilation] were abstracted by a trained research nurse independent of patient care. Statin intensity (high vs. moderate/none) and P2Y12 regimen at discharge were recorded. “Prior restenosis” indicated documented ISR/TLR in the target vessel before the index procedure.

### ISR ascertainment and imaging modalities

ISR at 12  ±  1 months was defined as ≥50% diameter stenosis within or ≤5 mm from the stent edge by invasive coronary angiography (ICA) or contrast-enhanced coronary CT angiography (CCTA). ICA assessments were performed by two interventional cardiologists blinded to biomarker data, using visual estimation corroborated by quantitative coronary angiography (QCA) when available. CCTA employed semiautomated lumen analysis with manual confirmation by two level III readers blinded to biomarkers and clinical covariates. Because CCTA ' lower specificity in stented segments, we prespecified an ICA-only sensitivity analysis to test the robustness of biomarker associations.

### Sample size calculation

We targeted enrollment of 2,500 patients to yield ≥200 ISR events, assuming an ISR incidence of ∼8% at 12 months. This ensures ≥10 events per parameter for multivariable logistic models with up to 20 degrees of freedom, in line with contemporary guidance for prognostic modeling. With an anticipated antiphospholipid antibody (aPL) prevalence of ∼12%, this sample provides 80% power (two-sided *α* = 0.05) to detect an adjusted odds ratio ≥1.9 for aPL positivity.

### Baseline medications and prior restenosis

At index admission/discharge, medications were abstracted from the electronic record. Statin intensity followed contemporary criteria: high-intensity vs. moderate/none. The DAPT regimen was categorized by P2Y12 inhibitor at discharge (clopidogrel vs. ticagrelor/prasugrel). Aspirin use was near-universal and not modeled. Prior restenosis was defined as a documented history of ISR or TLR in the target vessel before the index procedure.

### Blood sampling and biomarker assays

*Timing and processing*: Peripheral venous blood was drawn within 2 h before PCI into EDTA and 3.2% sodium-citrate tubes, centrifuged at 1,500 × *g* for 15 min at 4°C. Plasma and serum were aliquoted into bar-coded cryovials and snap-frozen at −80°C within 60 min.

*Antiphospholipid antibodies*: aCL IgG/IgM and anti-β2-glycoprotein I (anti-β2GPI) IgG/IgM were quantified by microplate ELISA (QUANTA Lite®, Inova Diagnostics/Werfen, San Diego, CA, USA), with calibrators traceable to GPL/MPL (aCL) and SGU/SMU (anti-β2GPI) units. Analytical ranges were 0–150 U with inter-assay CVs 5%–9% (IgG) and 6%–10% (IgM). Per prespecification, we defined positivity as ≥40 U or >99th percentile and additionally report titers in <20, 20–39, and ≥40 U bins. All aPL assays were performed once, on samples drawn within 2 h pre-PCI. Accordingly, aPL status reflects single-time seropositivity and does not establish APS classification. Because samples were obtained once pre-PCI, aPL status reflects single-time seropositivity and not APS classification, which requires persistent positivity at ≥12 weeks at medium/high titers (17).

*Lupus anticoagulant (LA)*: LA testing followed International Society on Thrombosis and Haemostasis Scientific and Standardization Committee (ISTH SSC) guidance for screening/confirm ratios, mixing studies, and demonstration of phospholipid dependence, with recommended mitigations for anticoagulated samples (18). LA testing also followed Clinical and Laboratory Standards Institute (CLSI) H60 recommendations. Briefly, platelet-poor plasma (<10  ×  10^9^/L) was prepared by double centrifugation of 3.2% sodium-citrate samples. Aliquots were frozen at −80°C and thawed once. Screening used dilute Russell viper venom time (dRVVT) with low-phospholipid screen and high-phospholipid confirm reagents (on an automated coagulation analyzer), with results expressed as ratios normalized to pooled normal plasma. A LA-sensitive aPTT (low-phospholipid formulation) was reflexed when dRVVT results were borderline/discordant. Mixing studies (1:1 patient–pooled normal plasma) were performed on prolonged screens. Phospholipid dependence was demonstrated by dRVVT confirm and, for borderline/discordant patterns, a hexagonal-phase/platelet phospholipid neutralization. LA was classified as positive when (1) screen ratio ≥1.20 (local 99th percentile-based cutoff); (2) no correction on mixing (mixing test ratio ≥1.20 or Rosner index of circulating anticoagulant >15); and (3) phospholipid dependence (normalized screen/confirm ratio ≥1.20 or ≥10% correction with high-phospholipid reagent/platelet neutralization procedure (PNP)). Results were negative if criteria were not met and uninterpretable when anticoagulant interference precluded reliable classification despite mitigations. The laboratory verified cutoffs in 120 healthy donors (mean  ±  2.3 SD/99th percentile approach) and participated in external proficiency testing; inter-assay CVs for dRVVT ratios were <5%.

*Rheumatic-immune inflammatory (RII) markers*: High-sensitivity C-reactive protein (hs-CRP) was determined by nephelometry (detection limit 0.15 mg/L). IL-6 and TNF-α were measured using a magnetic bead-based Luminex assay. Erythrocyte sedimentation rate (ESR) was determined by the Westergren method. Complement C3 and C4 were evaluated by immunoturbidimetric assay. Rheumatoid factor (RF) and anti-cyclic citrullinated peptide (anti-CCP) were measured by electrochemiluminescence. To minimize inter-assay variability, all IL-6 and hs-CRP measurements were performed in a single analytical batch using one reagent lot and single-thaw aliquots. Assays were run in duplicate, and the mean value was analyzed. Each plate included pooled-plasma quality control material (low and high), and runs were accepted only if inter-plate CVs were <10% for IL-6 and <5% for hs-CRP and if control values fell within predefined limits. When more than one microplate was required, they were processed on the same day under the same calibration, and pooled controls confirmed no material drift across plates.

Duplicate determinations were performed for all biomarkers; assays with a coefficient of variation (CV) of >10% were repeated. Laboratory staff were blinded to clinical outcomes.

### Management of anticoagulated patients

Per ISTH SSC guidance, testing was avoided whenever possible during active anticoagulation. When unavoidable, direct oral anticoagulant (DOAC) samples were processed with activated-charcoal adsorption (DOAC-Stop™/equivalent) and considered interpretable only if anti-Xa activity ≤ 0.1 IU/mL (for factor Xa inhibitors) or dilute thrombin time normalized (for dabigatran); vitamin K antagonist (VKA) samples were retested after interruption (international normalized ratio (INR) ≤ 1.5) whenever feasible. Heparin effects were mitigated using heparin-neutralizing reagents (validated to ∼0.8 IU/mL) and anti-Xa monitoring. Any sample failing these criteria was labeled “LA uninterpretable due to anticoagulation.”

### Statistical analysis

Analyses followed a prespecified statistical analysis plan and were performed in R (version 4.4). Continuous variables were presented as mean ± SD or median (IQR) and categorical variables as *n* (%). Between-group differences were assessed with Student's *t*-test or the Mann–Whitney *U* test for continuous variables and the *χ*² test or Fisher's exact test for categorical variables. Biomarker missingness was anticipated to be <5%; if missingness exceeded 2%, multiple imputation by chained equations (*m* = 20) was applied.

*Model performance and internal validation*: Discrimination was assessed by AUC (DeLong). Calibration was assessed by calibration slope/intercept, Hosmer–Lemeshow, Brier score, and bootstrap internal validation (*B*  =  1,000; optimism-corrected AUC and calibration). Decision curve analysis quantified net benefit across 2%–20% risk thresholds.

*Incremental prediction*: We compared a clinical model (traditional predictors only) vs. an immune-enhanced model (clinical  +  aPL  +  IL-6) using AUC difference (DeLong), categorical NRI (risk strata <5%, 5%–10%, >10%), and integrated discrimination improvement (IDI).

*ICA*-*only co*-*primary analysis*: We repeated the modeling restricted to participants assessed by ICA to evaluate robustness to modality-specific misclassification.

*Sensitivity analyses*: (1) Excluding participants with documented systemic autoimmune disease; (2) excluding LA-positive cases; (3) redefining ISR as ≥70% stenosis; (4) inverse probability weighting for incomplete imaging using a logistic model for the probability of completing 12-month imaging (predictors: age, sex, risk factors, presentation, stent parameters, and comorbidities) to assess potential selection bias; (5) adjustment or stratification by ACI score and IPTW for aortic calcification index (ACI)  >  0.

*Mediation analysis (exploratory/assumption*-*dependent)*: We estimated the average causal mediation effect (ACME) of IL-6 in the aPL to ISR pathway using nonparametric bootstrapping (5,000 resamples) with the same covariates as the primary model. Estimates (ACME/average direct effect (ADE)/TE, proportion mediated on the log-odds scale) are presented as exploratory, conditional on sequential ignorability (no unmeasured confounding of exposure–mediator and mediator–outcome relations, and no mediator measurement error).

*TLR analysis*: Time origin was the index PCI date. We generated Kaplan–Meier curves (log-rank tests) and fitted multivariable Cox models including the same covariates as logistic models (aPL, log_2_-IL-6, and clinical predictors). Patients were censored at last contact, non-TLR revascularization of another segment, death, or 365 days. Proportional hazard assumptions were checked by Schoenfeld residuals.

## Results

**Figure 1:**
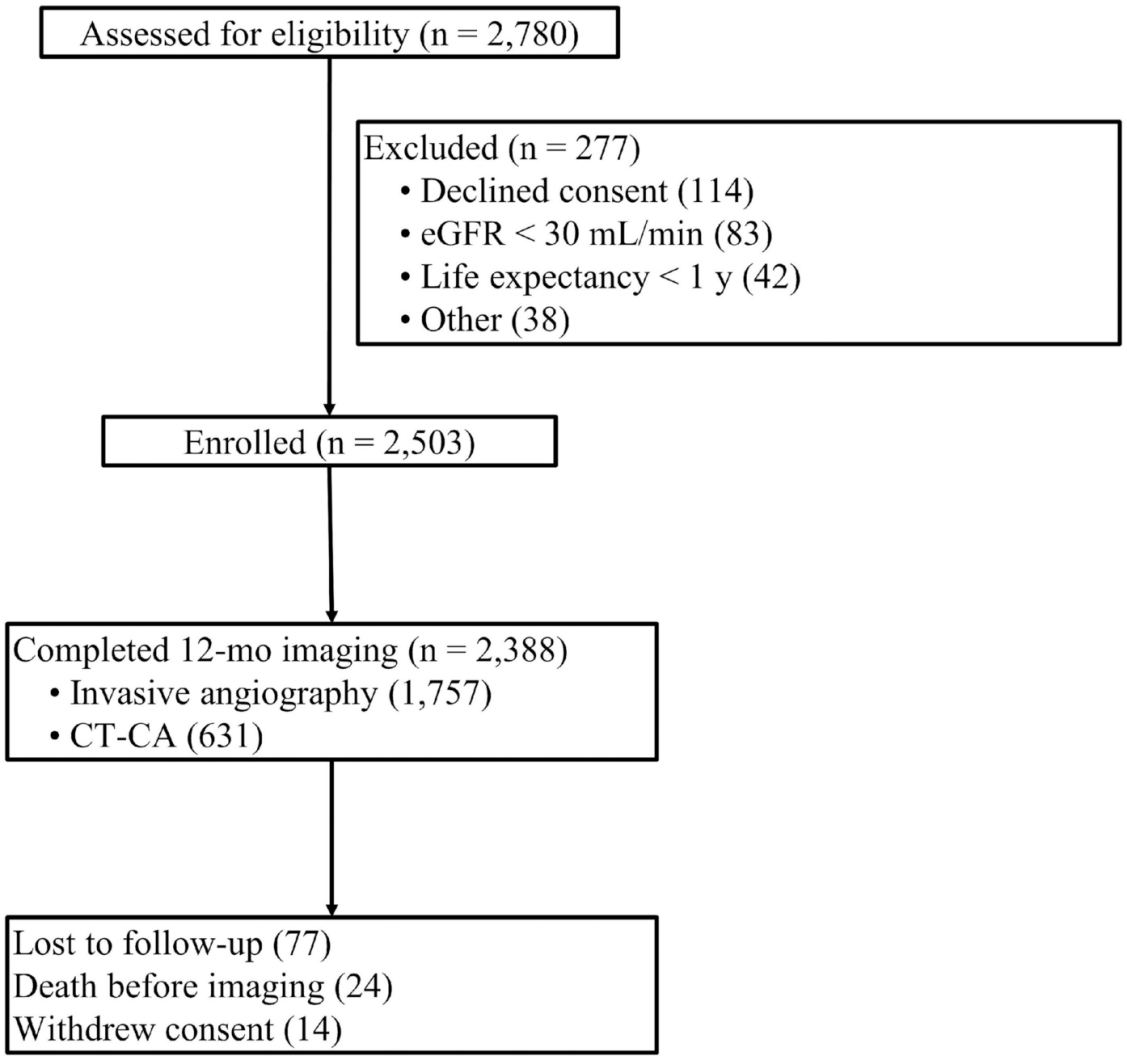
Study flow diagram. Stepwise screening and follow-up of the 2,780 patients assessed for eligibility. A total of 2,503 patients met the inclusion criteria and received new-generation drug-eluting stents; 2,388 completed 12-month imaging (1,757 invasive angiograms and 631 coronary CT angiograms). Reasons for exclusion and loss to follow-up are detailed in each box.

Of the 2,780 patients screened between May 2022 and January 2024, 2,503 met eligibility and were enrolled (Figure 1). A 12-month imaging was completed in 2,388 participants (1,757 invasive coronary angiography; 631 contrast-enhanced coronary CT angiography). Follow-up was incomplete in 115 patients due to loss to follow-up (*n* = 77), non-cardiac death (*n* = 24), or withdrawal of consent (*n* = 14).

The cohort's mean age was 62 ± 10 years, and 28.6% were women (Table 1). At 12  ±  1 months, ISR was confirmed in 193 of 2,388 patients (8.1%; 95% CI 6.9–9.1). Patients without 12-month imaging were not classified as “no ISR” and were excluded from ISR analyses. Diabetes mellitus was present in 20% overall and was more frequent among patients who developed ISR (32.1% vs. 19.0%; *p* < 0.001). Compared with those without ISR, ISR cases had longer treated lesions (28 ± 9 vs. 23 ± 7 mm) and smaller mean stent diameters (2.8 ± 0.3 vs. 3.0 ± 0.4 mm; both *p* < 0.001).

**Table 1:** Baseline characteristics and biomarker levels.

Variable	ISR (*n* = 193)	No ISR (*n* = 2,195)	*p*
Age (years), mean ± SD	64 ± 9	61 ± 10	0.01
Sex, *n* (%)			0.11
Male	147 (76.2%)	1,638 (70.9%)	
Female sex	46 (23.8%)	669 (29.1%)	
Diabetes, *n* (%)	62 (32.1%)	438 (19.0%)	<0.001
Current smoker, *n* (%)	81 (42.0%)	784 (33.9%)	0.03
LDL-C (mM), mean ± SD	3.0 ± 0.9	2.8 ± 0.9	0.02
Stent length (mm), mean ± SD	28 ± 9	23 ± 7	<0.001
Stent diameter (mm), mean ± SD	2.8 ± 0.3	3.0 ± 0.4	<0.001
Any aPL positive, %	46 (23.8%)	230 (9.96%)	<0.001
IL-6 (pg/mL), median [IQR]	4.7 (3.2–6.5)	2.2 (1.5–3.5)	<0.001
hs-CRP (mg/L), median [IQR]	3.4 (2.2–4.6)	1.8 (1.0–2.8)	<0.001

In modality-inclusive analyses of the imaging-complete cohort (Table 2), established angiographic predictors were significant (longer stent length, smaller stent diameter). Critically, any aPL positivity and higher IL-6 were each independently associated with ISR (aPL OR 1.92, 95% CI 1.34–2.74; IL-6 per doubling OR 1.25, 95% CI 1.10–1.42). hs-CRP attenuated in the fully adjusted model (OR 1.12; *p* =  0.09). These estimates reproduce your corrected calculations on the imaging-complete cohort and do not rely on classifying imaging-incomplete patients as “no ISR.”

**Table 2:** Multivariable logistic regression for 12-month ISR.

Predictor	Adjusted OR (95% CI)	*p*
Diabetes	1.42 (1.02 – 1.97)	0.044
Stent length (per 10 mm)	1.23 (1.11 – 1.36)	<0.001
Stent diameter (per 0.25 mm)	0.82 (0.71 – 0.94)	0.004
Lesion complexity (B2/C)	1.38 (0.99 – 1.93)	0.062
Any aPL-positive	1.92 (1.34 – 2.74)	<0.001
IL-6 (per doubling)	1.25 (1.10 – 1.42)	0.001
hs-CRP (per doubling)	1.12 (0.98 – 1.28)	0.086

**Figure 2:**
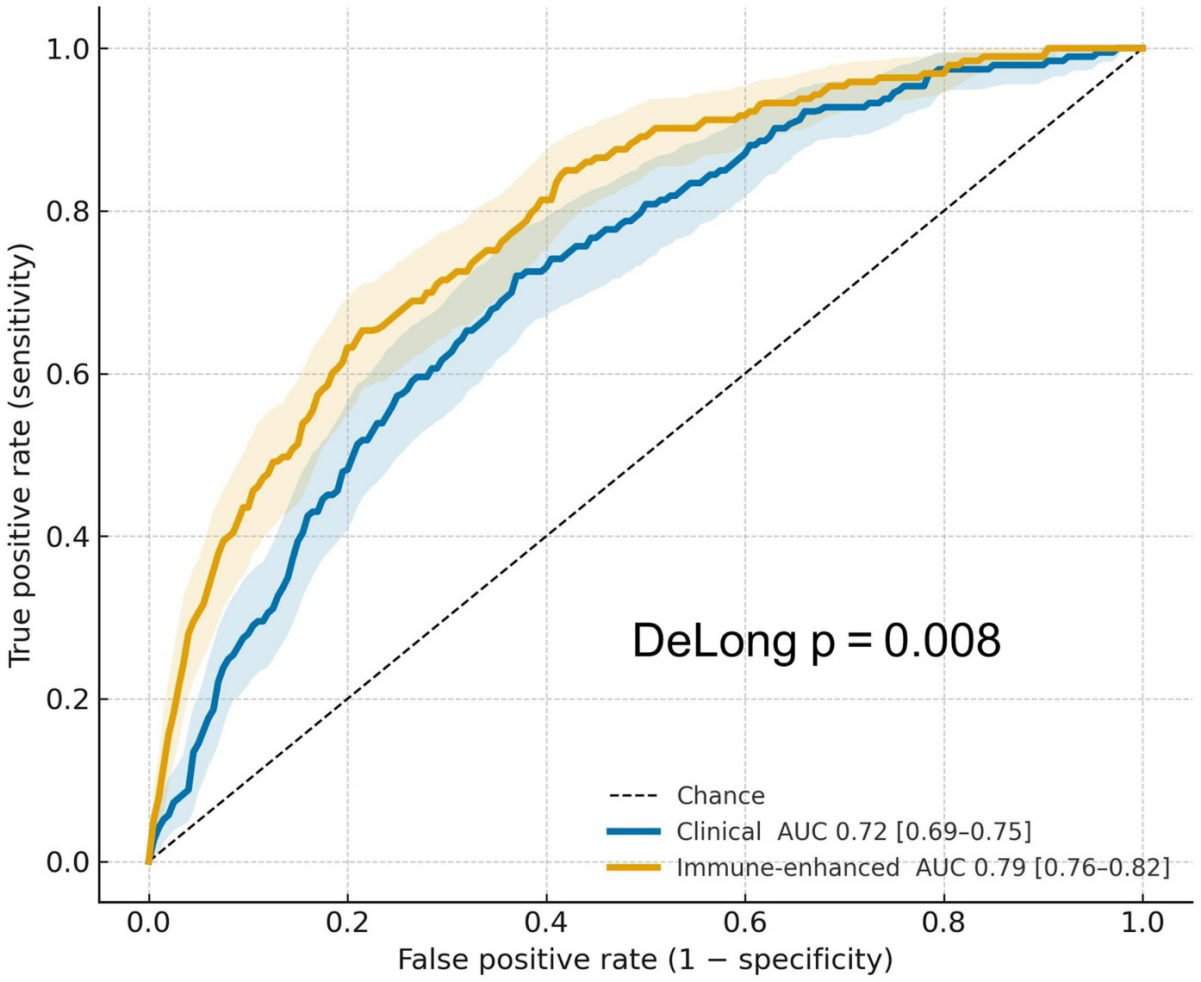
Receiver operating characteristic (ROC) curves for 12-month ISR prediction. ROC curves for the clinical and immune-enhanced models, with bootstrap 95% confidence bands (*B* = 2,000) and DeLong AUC (95% CI).

Adding aPL and IL-6 to the clinical model improved discrimination (AUC 0.79 vs. 0.72; Δ  =  0.07; *p* =  0.008), with better calibration (slope ≈ 1.01; Hosmer–Lemeshow *p* =  0.45) and a lower Brier score (0.058 vs. 0.064). Net reclassification (NRI  =  0.18, *p* =  0.01) and IDI  =  0.04 favored the immune-enhanced model. Bootstrap validation (*B*  =  1,000) showed minimal optimism (AUC 0.78 vs. 0.71 for the clinical model), supporting internal stability (Table 3 and Figure 2).

**Table 3:** Predictive performance of clinical vs. immune-enhanced models.

Metric	Clinical model	Immune model	*Δ*/*p*-value
AUC (95% CI)	0.72 (0.68–0.76)	0.79 (0.75–0.82)	+0.07; *p* = 0.008
Calibration slope	0.93	1.01	–
Hosmer–Lemeshow p	0.19	0.45	–
Brier score	0.064	0.058	–
Net reclassification improvement	–	0.18;	*p* = 0.01
Integrated discrimination improvement	–	0.04	–
Bootstrap AUC (optimism-corrected)	0.71	0.78	–

To address modality-specific misclassification, an ICA-only analysis (*n* =  1,757) showed consistent associations: aPL OR 1.95 (95% CI 1.24–3.05) and IL-6 OR 1.27 (95% CI 1.09–1.48) (Table 4). Results were also robust when ISR was redefined as ≥70% stenosis, when patients with systemic autoimmune disease were excluded, with IPTW for ACI  >  0, and after excluding LA-positive cases (Table 4). Isotype/titer analyses indicated that high-titer IgG aPL carried the strongest associations.

**Table 4:** Unified sensitivity analyses for ISR (logistic models).

Scenario	aPL OR (95% CI)	IL-6 OR (95% CI)
**ICA-only** (*n* = 1,757)	1.95 (1.24–3.05)	1.27 (1.09–1.48)
ISR ≥ 70% definition	2.10 (1.29–3.42)	1.35 (1.12–1.62)
Exclude systemic autoimmune disease	1.83 (1.24–2.70)	1.22 (1.07–1.40)
IPTW for ACI > 0	1.88 (1.28–2.73)	1.24 (1.09–1.41)
Exclude LA-positive	1.84 (1.23–2.76)	1.24 (1.09–1.42)

**Figure 3:**
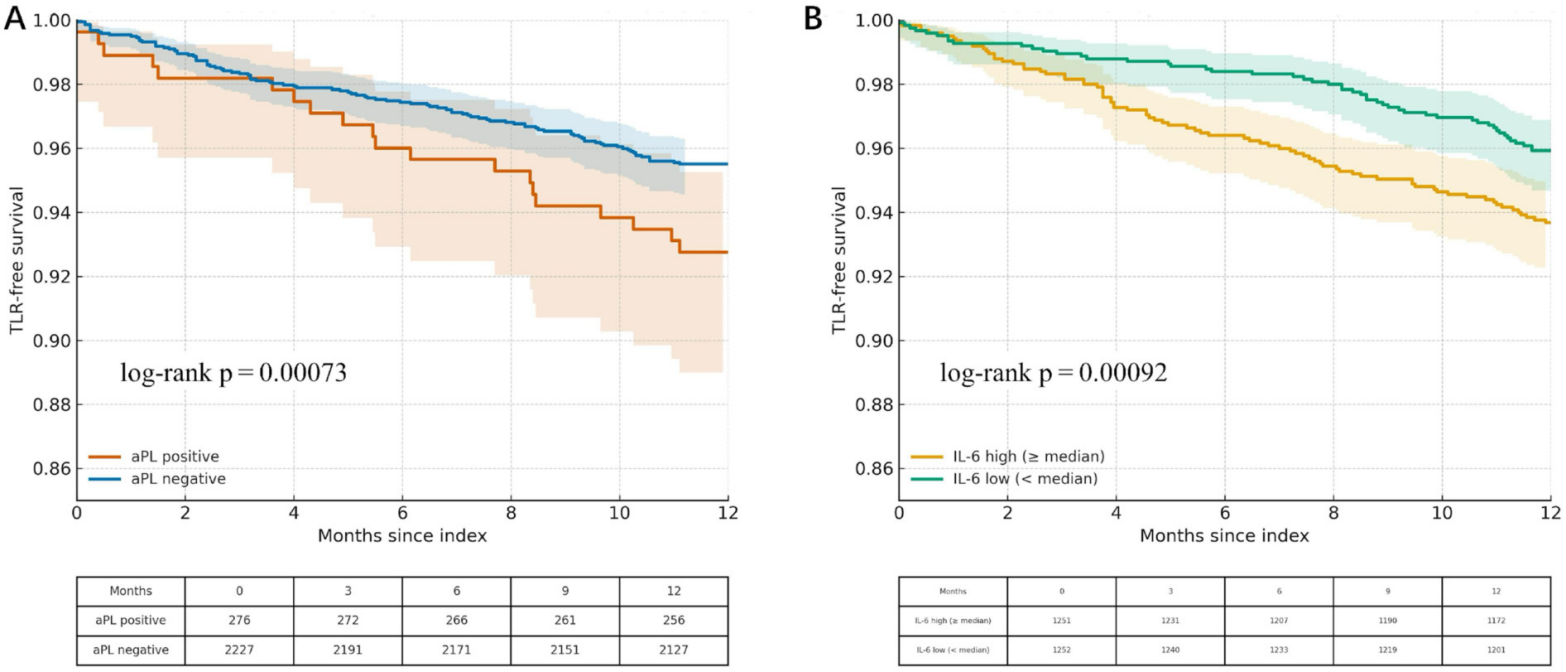
**(A)** Kaplan–Meier freedom from target-lesion revascularisation (TLR) by aPL status. Time-to-event curves for biomarker-positive (any aPL) vs. biomarker-negative patients over 365 days after PCI. Log-rank *p* < 0.001. **(B)** Kaplan–Meier freedom-from-TLR by IL-6 quartiles. Survival curves stratified by baseline IL-6 quartile (Q1 lowest to Q4 highest). A stepwise decline in TLR-free survival is evident with increasing IL-6 concentration (overall log-rank *p* < 0.001).

Over 12 months, 100/2,503 (4.0%) underwent TLR. Freedom from TLR was lower in aPL-positive patients and declined stepwise across IL-6 quartiles (Figure 3). In adjusted Cox models, aPL positivity (HR 2.08, 95% CI 1.36–3.18) and IL-6 (per doubling; HR 1.29, 95% CI 1.11–1.50) were independently associated with higher TLR risk (Table 5).

**Table 5:** Cox regression for 12-month TLR.

Predictor	Adjusted HR (95% CI)	*p*
Any aPL-positive	2.08 (1.36–3.18)	0.001
IL-6 (per doubling)	1.29 (1.11–1.50)	0.001
Diabetes	1.35 (0.98– 1.86)	0.07
Stent length (per 10 mm)	1.18 (1.05–1.33)	0.006
Stent diameter (per 0.25 mm)	0.83 (0.70 – 0.99)	0.04

## Discussion

Our findings align with contemporary summaries of the immune-coronary axis, in which inflammatory cytokines shape vascular healing and neointimal responses after PCI (19). In this large, prospective Chinese cohort that coupled comprehensive autoantibody testing with cytokine profiling in the era of new-generation DES, baseline aPL positivity and higher IL-6 were each independently associated with the risk of both ISR and clinically driven TLR. Adding these immune markers to a conventional clinical model improved discrimination. In mediation analysis, IL-6 accounted for approximately 27% of the total association between aPL and ISR, supporting an interlinked autoimmune-inflammatory pathway in restenotic healing.

Our findings confirm and extend earlier observations that autoimmunity increases the risk of coronary ISR. Small case reports and retrospective cohorts have consistently shown that patients with APS or SLE experience disproportionately high repeat-revascularization rates after PCI, even in the bare-metal stent era (20). More recent DES era data in RA and SSc similarly document excess ISR and adverse cardiac events (21, 22). In our DES-only cohort, baseline aPL positivity was associated with nearly twofold higher odds of 12-month ISR, in line with meta-analytic estimates in APS/SLE and demonstrating that this risk persists despite contemporary stent technology. The inflammatory milieu appears central. Whereas earlier meta-analyses linked elevated CRP to post-PCI events (23), our multivariable model—and others (24)—identify IL-6 as the stronger ISR predictor, with incremental discrimination comparable to gains achieved by adding HbA1c or on-treatment platelet reactivity in prior predictive modeling studies (25).

Mechanistically, aPL bind β2-glycoprotein I (β2GPI) on endothelial cells, upregulating ICAM-1/VCAM-1, triggering complement activation (C3, C5), and engaging the mTOR pathway processes that promote thrombosis and vascular smooth-muscle cell (VSMC) proliferation (26–30). *In vitro*, aPL enhances endothelial migration and matrix metalloproteinase-9 activity, whereas complement split products amplify IL-6 release and downstream Th17 responses, phenomena well documented in APS and SLE (31, 32). IL-6 is a potent amplifier of cytokine cascades that drives VSMC migration and extracellular matrix deposition; notably, IL-6 blockade attenuates neointimal hyperplasia in NZB/W F1 lupus-prone mice (14). Our mediation analysis, showing that IL-6 accounted for ∼27% of the aPL effect on ISR, fits this biology, while the remaining ∼73% direct effect suggests additional mechanisms, such as fibrin-rich microthrombi or immune-complex deposition within restenotic tissue (12). These mediation estimates are assumption-dependent and exploratory. Given single-time point biomarkers and potential measurement error, the ∼27% figure should be treated as descriptive on the log-odds scale, not as evidence that IL-6 explains the effect.

Although aPL and IL-6 were measured pre-PCI, and ISR was ascertained 12 months later, reducing the risk that the outcome directly influenced biomarker levels, reverse causation cannot be entirely excluded. Systemic inflammation could predispose to both higher biomarker levels and restenosis propensity. We adjusted for clinical covariates and hs-CRP and performed sensitivity analyses with unchanged inferences. Nevertheless, residual confounding from unmeasured inflammatory or immunologic factors remains possible. Consistent with this, we frame the findings as associations rather than causal effects, and the mediation results as assumption-dependent and hypothesis-generating.

Clinically, these findings support integrating immune inflammatory profiling into routine PCI practice. Validation data suggest that a preprocedural panel combining aPL, IL-6, and hs-CRP improves ISR risk prediction, particularly in APS and RA populations (25, 33), and the SII provides complementary prognostic information in acute coronary syndromes and diabetes (25, 34, 35). High-risk patients could undergo earlier surveillance to detect subclinical restenosis (36). Therapeutically, IL-6 blockade (e.g., tocilizumab), low-dose colchicine, or preferential use of sirolimus-eluting (mTOR-inhibiting) stents may mitigate immune-driven ISR; ongoing anti-cytokine trials in CAD offer a framework for testing these strategies (33, 35). Finally, in aPL-positive patients with demonstrable thrombophilia, extended dual antiplatelet therapy or adjunctive low-dose anticoagulation warrants prospective evaluation to personalize secondary prevention after PCI.

Nevertheless, several limitations merit consideration. As an observational study, residual confounding cannot be excluded. Biomarkers were measured at a single baseline time point, precluding assessment of post-PCI dynamics. Finally, this single-center cohort may limit generalizability. aPL was measured at a single baseline time point; thus, our findings reflect aPL serostatus rather than APS classification, which requires persistent positivity at ≥12 weeks at medium/high titers. Moreover, transient elevations in aPL can occur with intercurrent infection, recent vaccination, or acute illness, which we did not systematically adjudicate. Some misclassification toward transient positivity is therefore possible. ISR was ascertained by both ICA and CCTA. Although CCTA enables standardized follow-up, specificity is lower in stented segments due to blooming artifacts, small-lumen segmentation challenges, and heavy calcification; any such misclassification is expected to be nondifferential with respect to aPL/IL-6, biasing associations toward the null. Future work should externally validate the immune-enhanced prediction model across diverse populations; incorporate serial measurements of IL-6 and aPL to determine whether post-procedural trajectories further refine risk stratification; and pair biomarker profiling with mechanistic imaging to link immune signatures with neointimal phenotypes. Randomized trials of targeted anti-inflammatory or immunomodulatory therapies—such as IL-6 blockade or colchicine—in biomarker-positive patients, alongside genomic and epigenomic studies to identify susceptibility loci for immune-mediated ISR, will be essential to translate these findings into precision cardiovascular care.

In summary, antiphospholipid antibodies and heightened interleukin 6-mediated inflammation are independently associated with drug-eluting stent failure, increasing the risks of restenosis and clinically driven repeat revascularization despite contemporary PCI practice and supporting further development of immune-enhanced risk stratification and secondary prevention for ISR.

## Data Availability

The raw data supporting the conclusions of this article will be made available by the authors, without undue reservation.
